# Supplementary material for: Content-rich biological network constructed by mining PubMed abstracts
Source: BMC Bioinformatics. 2004 Oct 8;5:147. doi: 10.1186/1471-2105-5-147 (PMC528731; doi:10.1186/1471-2105-5-147)
Supplement: Additional File 2 — The original results of the above study (non-essential files are deleted to keep the file size under the limit set by BMC bioinformatics). [file 1471-2105-5-147-S2.bz2 › chilibotAdditionalFile2/dip30/444ID9468473E10421/html/CCL3_CCR5.html]

 


 **CCL3** and **CCR5** 
  
Found 94 abstracts in PubMed, retrieved 30.  
 

 What does Google say? 
 PDF only 
| .edu only 

---

**Interactive relationship** (e.g. stimulation, inhibition, etc)

**Neutral relationship**- Strong interaction with  **CCR5**  means that  **CCL3**  L1, and particularly its 2 variant, are by far the most potent natural HIV entry inhibitors described to date.  Ref: 12355456 Eur J Immunol, 2002
- Although virus specific CD8 T cells from  **CCL3**  mice were CD44 high, they remained CD62L high and CD25 low, retained CCR7 expression, and contained limited transcripts of the proinflammatory chemokine receptors  **CCR5**  and CXCR3 compared with virus specific CD8 T cells from  **CCL3**  mice.  Ref: 12634360 J Virol, 2003
- However, the remaining expression of CCR1 and  **CCR5**  correlated reciprocally with an ongoing unresponsiveness to the CCR1 and  **CCR5**  agonists  **CCL3**  macrophage inflammatory protein 1alpha MIP 1alpha, CCL4 MIP 1beta, and CCL5 regulated on activation, normal T expressed and secreted RANTES, all chemokines binding to these two receptors.  Ref: 12885942 J Leukoc Biol, 2003
- We and others have shown that the human beta chemokine  **CCL3**  L1, unlike its related non allelic isoform  **CCL3** , has high affinity for the chemokine receptors D6, CCR3 and  **CCR5** .  Ref: 12355456 Eur J Immunol, 2002
- High levels of  **CCL3**  and CCL4 were produced within 8 h after infection, which is likely to account for the observed  **CCR5**  down modulation on Mtb infected DC.  Ref: 12538673 J Immunol, 2003
- **CCR5**  is a G protein coupled receptor responding to four natural agonists, the chemokines RANTES regulated on activation normal T cell expressed and secreted, macrophage inflammatory protein MIP 1 alpha  [ **CCL3** ] , MIP 1 beta, and monocyte chemotactic protein MCP 2, and is the main co receptor for the macrophage tropic human immunodeficiency virus strains.  Ref: 12411445 J Biol Chem, 2003
- Our studies showed that LD infection caused the upregulation of three beta chemokines macrophage inflammatory protein 1 alpha  [ **CCL3** ]  MIP 1alpha, MIP 1beta and RANTES regulated on activation normal T cell expressed and secreted, one alpha chemokine interleukin 8 IL 8 and the CC chemokine receptor 5  [ **CCR5** ]   **CCR5**  but not CCR1, as evident from reverse transcriptase polymerase chain reaction RT PCR analysis.  Ref: 12662300 Scand J Immunol, 2003
- Supernatants from samples with high copy number are able to more potently chemoattract  **CCR5**  expressing cells, an effect blocked with anti  **CCL3**   **CCL3**  L1 antibodies.  Ref: 12355456 Eur J Immunol, 2002
- The MIP 1 alpha  [ **CCL3** ]  isoform LD78 beta, that was purified form natural sources, inhibited HIV infection completely in  **CCR5**  transfected cells, mononuclear leukocytes and purified monocytes at low ng ml concentrations.  Ref: 12649932 Verh K Acad Geneeskd Belg, 2002
- Moreover,  **CCL3**  L1, but not  **CCL3** , is susceptible to cleavage by CD26, creating a truncated 2 form with enhanced affinity for CCR1 and  **CCR5** .  Ref: 12355456 Eur J Immunol, 2002
- The higher RANTES production by MS gamma delta T cell lines, together with a lower expression of  **CCR5** , may reflect an autoregulatory loop, caused by an increased production of its ligands RANTES, MIP 1 alpha  [ **CCL3** ] , and MIP 1 beta or due to other pro inflammatory cytokines.  Ref: 12173306 Clin Immunol, 2002

**Non-interactive relationship** (e.g. studied together, co-existance, homology, etc.)

- The  **CCR5**  ligands, RANTES and MIP 1 alpha  [ **CCL3** ] , are expressed in the spinal cords of mice at EAE onset.  Ref: 12667654 J Neuroimmunol, 2003
- To test the in vivo relevance of this, we used a murine melanoma system and knockout mice to investigate the function of the chemokine receptor  **CCR5**  and its ligands, CCR ligand CCL 3  [ **CCL3** ]  and CCL5.  Ref: 12682253 J Immunol, 2003
- Bacterial LPS protects primary human macrophages from infection by  **CCR5**  tropic HIV 1 isolates through the release of the CC chemokines RANTES and macrophage inflammatory protein 1 alpha  [ **CCL3** ]  and 1 beta.  Ref: 12055257 J Immunol, 2002
- Since both human MIP 1alpha subtypes LD78alpha  [ **CCL3** ]  and LD78beta and MIP 1beta signal through  **CCR5** , the major co receptor for M tropic HIV 1 strains, these chemokines are capable of inhibiting HIV 1 infection in susceptible cells.  Ref: 12401480 Cytokine Growth Factor Rev, 2002
- In parallel, CCR1 and  **CCR5**  expression is down regulated and CXCL8, CCL2,  **CCL3**  and CCL5 production is up regulated.  Ref: 12548563 Eur J Immunol, 2003
- The expression of the chemokines macrophage inflammatory protein 1 alpha  [ **CCL3** ]  MIP 1alpha and interferon gamma inducible protein 10 IP 10 and of their respective receptors,  **CCR5**  and CXCR3, were more prevalent and higher in aggressive periodontitis, and associated with higher interferon gamma IFN gamma expression and lower interleukin 10 IL 10 expression.  Ref: 12608917 J Periodontal Res, 2003
- We also determined that  **CCR5**  binding chemokines MIP 1alpha  **CCL3** , CCL4, and RANTES CCL5, specifically by signaling through  **CCR5** , could affect all progenitor B cell populations through a novel mechanism involving heterologous desensitization of CXCR4.  Ref: 12239139 Blood, 2002
- In contrast, the loss of the  **CCR5**  ligand,  **CCL3** , led to an early delay in tumor growth that did not persist, while the absence of the  **CCR5**  ligand, CCL5, had no effect.  Ref: 12682253 J Immunol, 2003
- After 2 to 4 weeks, L. amazonensis infected mice had significantly delayed and depressed expression of inflammatory cytokines interleukin 12 IL 12, gamma interferon, IL 1alpha, IL 1beta, CC chemokines CC chemokine ligand 3  [ **CCL3** ]   **CCL3**  macrophage inflammatory protein 1alpha MIP 1alpha, CCL4 MIP 1beta, CCL5 RANTES, MIP 2, and chemokine receptors CCR1, CCR2,  **CCR5**  in foot tissues and draining lymph nodes compared to the expression in L. major infected controls.  Ref: 12874303 Infect Immun, 2003
- A number of inflammatory CC chemokines, including MIP 1 alpha  [ **CCL3** ] , MIP 1 beta, RANTES, MCP 2, and HCC 1 9 74 act as  **CCR5**  agonists, while MCP 3 is a natural antagonist of the receptor.  Ref: 12402506 Receptors Channels, 2002
- In contrast,  **CCL3** , a ligand of CCR1 and  **CCR5** , induced rapid uptake of FITC dextran by immature DCs but not mature DCs.  Ref: 12609829 Blood< MedlineTA>Blood, 2002
